# Cumulative physiological stress is associated with age-related changes to peripheral T lymphocyte subsets in healthy humans

**DOI:** 10.1186/s12979-023-00357-5

**Published:** 2023-06-23

**Authors:** Ryan G. Snodgrass, Xiaowen Jiang, Charles B. Stephensen, Kevin D. Laugero

**Affiliations:** 1 Immunity and Disease Prevention Research Unit, United States Department of Agriculture-Agricultural Research Services, Western Human Nutrition Research Center, 430 West Health Sciences Drive, Davis, CA 95616 USA; 2 Obesity and Metabolism Research Unit, United States Department of Agriculture-Agricultural Research Services, Western Human Nutrition Research Center, Davis, CA USA; 3 Department of Nutrition, University of California Davis, Davis, CA USA

**Keywords:** Regulatory T lymphocyte, Stress, Allostatic load, Treg, Allostasis, Immunosenescence, Aging

## Abstract

**Background:**

Progressive age-associated change in frequencies and functional capacities of immune cells is known as immunosenescence. Despite data linking chronic environmental, physiological, and psychosocial stressors with accelerated aging, how stress contributes to immunosenesence is not well characterized.

**Objective:**

To help delineate the contribution of cumulative physiological stress on immunosensence we assessed relationships between a composite measurement of cumulative physiological stress, reflecting the functioning of the hypothalamic-pituitary-adrenal axis, sympathetic nervous system, cardiovascular system, and metabolic processes, and lymphocyte changes typically affiliated with aging in a cohort of healthy volunteers ranging from 18 to 66 y.

**Results:**

Physiological stress load positively correlated with subject age in the study cohort and was significantly higher in adults 50–66 y compared to adults 18–33 y and 34–49 y. Using physiological stress load, we identified a significant age-dependent association between stress load and frequencies of circulating regulatory T lymphocytes (Tregs). Frequencies were higher in younger participants, but only in participants exhibiting low physiological stress load. As stress load increased, frequencies of Tregs decreased in young participants but were unchanged with increasing stress load in middle and older age individuals. Follow-up analysis of stress load components indicated lower circulating DHEA-S and higher urinary norepinephrine as the primary contributors to the effects of total stress load on Tregs. In addition, we identified age-independent inverse associations between stress load and frequencies of naïve Tregs and naïve CD4 T cells and positive associations between stress load and frequencies of memory Tregs and memory CD4 T cells. These associations were primarily driven by stress load components waist circumference, systolic and diastolic blood pressure, CRP, and HbA1c. In summary, our study results suggest that, in younger people, physiological stress load may diminish regulatory T cell frequencies to levels seen in older persons. Furthermore, independent of age, stress load may contribute to contraction of the naïve Treg pool and accumulation of memory Treg cells.

**Clinical trial:**

Registered on ClincialTrials.gov (Identifier: NCT02367287).

**Supplementary Information:**

The online version contains supplementary material available at 10.1186/s12979-023-00357-5.

## Introduction

Aging is a natural and inevitable physiological process leading to the progressive decline in the function of cells, tissues, organs and organisms. As with other tissues and systems, the aged immune system is characterized by progressive dysfunction affecting the composition, quantity, and function of immune organs and immune cells collectively referred to as immunosenescence [1]. This age-associated decline in immune function is ultimately responsible for elevated susceptibility to severe bacterial and viral infections and decreased vaccination efficacy [1]. While age-related changes have been documented in both innate and adaptive arms of the immune system, changes are more pronounced in cells of the adaptive immune system comprised of B and T lymphocytes [2, 3]. One of the primary causes of immunosenescence in the T cell compartment is the involution of the thymus which is responsible for generating new and highly diverse naïve T cells from precursor originating within the bone marrow [2]. Reduced thymic output eventually leads to contraction of the naïve T cell pool and accumulation of highly differentiated memory T cells. Additional causes of T cell aging include mitochondrial dysfunction and genetic and epigenetic alterations resulting in reduced T cell receptor diversity and loss of effector cell plasticity thereby compromising the capacity of the immune system to respond to new antigenic challenges [2].

Whereas chronological aging increases at the same rate for everyone, biological aging is variable and is vulnerable to age-accelerating and -decelerating factors. While the response to intrinsic and extrinsic stressors is not typically thought of as a mechanism of aging, accumulating data suggests the biological stress response determined by a multiplicity of genetics, environmental, and developmental factors can actively shape the rate of aging [4, 5]. In contrast to an acute stress response which is essential for healthy functioning, chronic stress can accelerate aging, increase disease susceptibility, and even have long-lasting influence on the immune system [6, 7]. In an attempt to understand the consequences of chronic stress on the aging process, McEwan and Stellar introduced the concept of allostatic load (AL) in 1993 [8] as a measure of the cumulative physiological burden, or “wear and tear” exacted on the body over time through repeated attempts to adapt to life’s demands. The notion of AL relies on biological parameters reflecting functioning of the hypothalamic-pituitary-adrenal (HPA) axis, sympathetic nervous system (SNS), cardiovascular system, and metabolic processes. While none of the individual components exhibit strong predictive capacity, the composite AL score has been shown to be a significant predictor of cumulative biological risk and thus provides an intermediate phenotype of aging [9–11].

Relationships between stress and immune function are well described [6, 12]. Chronic stress mediated by SNS activation and the HPA axis modifies circulating leukocytes through increased bone marrow hematopoiesis [13], alters levels of various cytokines including CRP, IL-1β, IL-6, and TNF-a, and can induce neuroinflammation driven by microglial activation [6]. Despite data linking chronic stress with accelerated aging, how chronic environmental, physiological, and psychosocial stressors contribute to immunosenesence has not been well characterized. Therefore, in attempt to delineate the contribution of chronic physiological stress on age-related shifts in the immune system, we investigated the relationship between cumulative physiological stress and lymphocyte changes typically affiliated with immunosenescence in a cohort of healthy volunteers.

## Methods

### Study participants

Study participants were from the USDA Nutritional Phenotyping Study which included healthy men and women, aged 18–66 y with a normal to obese BMI of 18–44 kg/m^2^ living near Davis, California beginning in May 2015. Men and women were recruited to fill nine bins within sex, to balance BMI and age, using three BMI categories (< 25, 25 to 29, and 30 to 44 kg/m2) within each age category (18 to 33, 34 to 49, and 50 to 65 y). Participants were excluded if they had high blood pressure (systolic blood pressure greater than 140 mm Hg or diastolic blood pressure greater than 90 mm Hg) when measured on-site or if they had any active chronic disease requiring daily medication, including, but not limited to, diabetes mellitus, cardiovascular disease, cancer, gastrointestinal disorders, kidney disease, liver disease, bleeding disorders, asthma, autoimmune disorders, hypertension, or osteoporosis. Participants were also excluded if they were pregnant or lactating, had recently undergone minor surgery, recently received antibiotic therapy, had been hospitalized in the past 4 weeks or had major surgery in the past 16 weeks. Additional details of study recruitment, participation, and subject ethnicity are contained in separate reports [14–16]. The study was registered at clinicaltrials.gov (identifier: NCT02367287) and received ethical approval from the University of California, Davis, Institutional Review Board. All participants provided written informed consent and received monetary compensation for their participation. Data were stored using the Research Electronic Data Capture (REDCap) application hosted by the University of California Davis Health System Clinical and Translational Science Center.

### Calculation of physiological stress load (allostatic load)

Physiological stress load score was created using a method following the methodology described in the McArthur studies of successful aging [9–11] and that reported by Gallo et al. [17], which expands on the McArthur studies method and takes into account hyper and hypocortisolemia, as estimated by cortisol concentrations falling into the highest or lowest octiles (12.5%) of the study sample. Physiological stress load (allostatic load) was derived from resting systolic and diastolic blood pressure, waist circumference, 12-h overnight urinary cortisol, norepinephrine, and epinephrine levels (corrected for urinary creatinine levels), fasting serum levels of high sensitivity C-reactive protein (hs-CRP), total cholesterol, and HDL cholesterol, fasting plasma levels of dehydroepiandrosterone sulfate (DHEA-S), and whole blood hemoglobin A1c (HbA1c). The sample-specific empirical method was used to define cut-points for the score as follows: each parameter except cortisol was divided into quartiles and, except HDL cholesterol and DHEA-S, values falling within the top quartile were scored with one point. For HDL cholesterol and DHEA-S, one point was given for those values falling within the bottom quartile. As noted, cortisol was divided into octiles, and values that fell within the top or bottom octiles were assigned one point. An 11-item index was constructed such that each parameter was worth 1 point. Therefore, a higher score reflected a higher physiological stress load or worse health risk. This integrative biomarker incorporates subclinical measures (e.g., hs-CRP) across a range of multiple biomarkers that interact with activity in stress pathways including the SNS and HPA axis [18, 19].

### Clinical parameters and stress load components

Fasting blood was collected and serum or plasma was obtained by centrifugation at 1300 x g at 4 °C for 10 min. Total cholesterol and HDL-cholesterol (HDL-C) were measured using a Cobas Integra 400/800 kit (Roche, Indianapolis, IN), a Cobas CHOL2 kit (Roche), a Cobas HDL-C plus 3rd generation kit (Roche), respectively. All assays were completed on an auto-analyzer, Cobas Integra 400 + instrument (Roche). Urinary cortisol was measured using a Urinary Cortisol ELISA (Alpco Diagnostics, Salem, NH). Urinary epinephrine and norepinephrine were measured using the Bi-Cat Urine ELISA (Eagle Biosciences, Nashua, NH). Urine creatinine, total and HDL cholesterol, and HbA1c were determined with Roche reagents on the Integra 400 Plus clinical chemistry analyzer (Roche Diagnostics, Indianapolis, IN). CRP (high sensitivity) was measured using the Meso Scale Diagnostics (MSD) VIP2 (vascular injury panel 2) kit with the MSD Sector Imager 2400 and SQ 120 electrochemiluminescence instruments (Rockville, MD). DHEA-S was measured using Roche reagents on the Roche e411 electrochemiluminescence clinical chemistry analyzer. Waist circumference was measured as the minimum circumference between the iliac crest and the rib cage. Blood pressure was measured using a standard blood pressure cuff placed on one arm.

### Lymphocyte analysis by flow cytometry

Lymphocytes in fasting peripheral blood were analyzed by three flow cytometry panels (A-C) using isolated PBMCs as previously described [20]. PBMCs were resuspended in Brilliant Stain Buffer (BD Biosciences, San Jose, CA, USA) with one million PBMCs for each panel. PBMCs were stained with the Fixable Viability stain 510 (BD Biosciences) on ice prior to staining with the rest of the antibodies. Panel A - naïve and central/effector memory T-cells, with activation markers CD38 and HLA-DR; Panel B - Th1, Th2, Th17 Cells, NK cells and B cells; Panel C – total, naïve and memory Treg cells with activation markers CD38 and HLA-DR. Cells were analyzed using an LSRFortessa flow cytometer (BD Biosciences) configured with blue (488 nm), red (640 nm), violet (405 nm) and UV lasers (355 nm). Data were collected using FACSDiva and analyzed using FlowJo version 10.6.1 software (BD Biosciences). Antibodies (Supplementary Table 1) are available in the supplementary material. The gating strategies for panels A-C can be found in our previously published USDA Nutritional Phenotyping Study manuscript (PMID: 36248784).

### Statistical analysis

Statistical analyses were performed using SAS for Windows, release 9.4 (Cary, N.C.) and GraphPad Prism 9, version 9.5.0. The General Linear Models (GLM) procedure was used to test overall associations between physiological stress load and lymphocyte subset, and for physiological stress load by age interaction. The basic model was: lymphocyte subset = physiological stress load + age + physiological stress load*age. To determine the nature of a stress load by age interaction, we followed up with linear regression analysis in GraphPad to examine associations between stress load and lymphocyte marker at 3 categories of age; young (18–33 y), middle (34–49 y), and old (50–66 y). Using the same statistical approach, when a statistically significant effect of stress load or stress load by age interaction was observed, we also followed up to examine each of the stress load component variables (e.g., urinary cortisol). An example of the basic model for this analysis was: T lymphocyte marker = urinary cortisol + age + urinary cortisol*age. For this follow up analysis, we used the octile scores for each of the components. Stress load component variables and cell subset frequencies were compared between age groups using Kruskal-Wallis non-parametric one-way ANOVA with Dunn’s multiple comparisons test. For all statistical analyses a P-value of ≤ 0.05 was considered statistically significant (*P < 0.05; **P < 0.01; ***P < 0.001; ns = not significant). Final assembly and preparation of all figures were done using CorelDRAW 2021 (Corel Corporation, Ottawa, Canada).

## Results

### Relation of physiological stress load with subject age

**Fig. 1:**
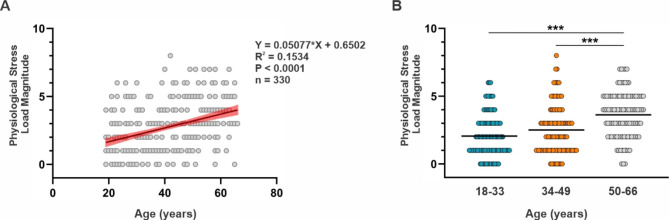
Physiological stress load in study cohort of healthy adults. (**A**) Relationship between subject age and physiological stress load. Black line indicates the linear line of best fit and red indicates the 95% confidence bands of the best-fit line. Equation, correlation squared (R^2^), *P* value for the association constant, and number of subjects are shown for the linear regression. (**B**) Physiological stress load grouped by subject age. Statistical analysis was performed using Kruskal-Wallis non-parametric one-way ANOVA; ***P < 0.001

To investigate the relationship between age-related changes in the adaptive immune system and cumulative physiological stress in a cohort of healthy adult volunteers ranging from 18 to 66 y, we first calculated a composite measure of each subject’s cumulative physiological stress load using the AL battery of biomarkers devised by Seeman and McEwen [8, 9, 11]. As shown in Fig. 1A, physiological stress load positively correlated with subject age in our cohort of healthy subjects. When stratified by age group (18–33 y, 34–49 y, and 50–66 y), adults 50–66 y exhibited a significantly higher physiological stress load compared to adults 18–33 y and 34–49 y (Fig. 1B). Physiological stress load parameters and mean values of each component used to calculate physiological stress load are presented in Table 1.

**Table 1:** Measurements of physiological stress load parameters in study cohort. Mean and range (in parenthesis) of quantitative parameters used to calculate physiological stress load. Kruskal-Wallis non-parametric one-way ANOVA was used for evaluating significance. Means not sharing a common letter are significantly different. P < 0.05 was considered significant. NS, not significant

		Age Groups (years)			
		18–33	34–49	50–66	Significance
	[n]	112	111	107	
Male/Female	[n]	55/57	58/53	47/60	
Age	[years]	24.82 (19–33)	40.95 (34–49)	56.99 (50–66)	
BMI	[kg/m^2^]	26.86 (18.21–38.68)	27.57 (18.04–43.25)	26.76 (19.09–40.08)	NS
**Physiological stress load**		2.054 (0–6)**a**	2.505 (0–8)**a**	3.626 (0–7)**b**	P < 0.0001
urinary Cortisol/Creatinine	[ug/g]	77.65 (20.95–320.3)**a**	94.30 (31.54–282.6)**b**	104.50 (26.18–356.6)**b**	P < 0.0001
urinary Epinephrine/Creatinine	[ug/g]	2.64 (0.33–12.31)	2.46 (0.21–12.22)	2.55 (0.31–12.07)	NS
urinary Norepinephrine/Creatinine	[ug/g]	22.4 (2.58–80.39)**a**	19.2 (3.08-75.00)**a**	28.6 (2.23–90.95)**b**	P < 0.0001
Waist circumference	[cm]	81.66 (60.8-120.2)**a**	86.96 (63.5-135.8)**b**	85.81 (66.7-115.2)**b**	P = 0.0054
Systolic blood pressure	[mmHg]	117.3 (93.5-138.5)**a**	119.2 (92.0-140.3)**ab**	122.3 (93.0-147.5)**b**	P = 0.0039
Diastolic blood pressure	[mmHg]	66.3 (50.0–85.0)**a**	69.8 (50.0-91.3)**b**	68.5 (51.0-93.5)**ab**	P = 0.0174
serum Cholesterol	[mg/dL]	160.3 (88.5-261.6)**a**	175 (118.8-263.1)**b**	191.7 (108.6-316.8)**c**	P < 0.0001
serum HDL Cholesterol	[mg/dL]	55.19 (27.5-109.4)	53.59 (23.8-108.3)	57.69 (27.9–116.0)	NS
plasma HbA1c	[%]	5.18 (4.6-6.0)**a**	5.32 (4.7–10.9)**b**	5.40 (4.8–6.5)**c**	P < 0.0001
plasma hs-CRP	[ng/mL]	4096 (62-75352)	3451 (50-40854)	3814 (127-32686)	NS
plasma DHEA-S	[ug/dL]	310.9 (107.7–1000.0)**a**	223.6 (30.0-497.3)**b**	139.0 (33.9-378.4)**c**	P < 0.0001

**Abbreviations**: DHEA-S, dehydroepiandrosterone sulfate; HbA1c, hemoglobin A1c; hs-CRP, high sensitivity c-reactive protein.

The mean and range (in parenthesis) is given for quantitative parameters. Kruskal-Wallis non-parametric one-way ANOVA was used for evaluating significance and Dunn’s multiple comparisons test was used to compare groups. Means not sharing a common letter are significantly different. P < 0.05 was considered significant. NS, not significant.

### Relation of physiological stress load with age-associated changes in regulatory T lymphocytes

**Fig. 2:**
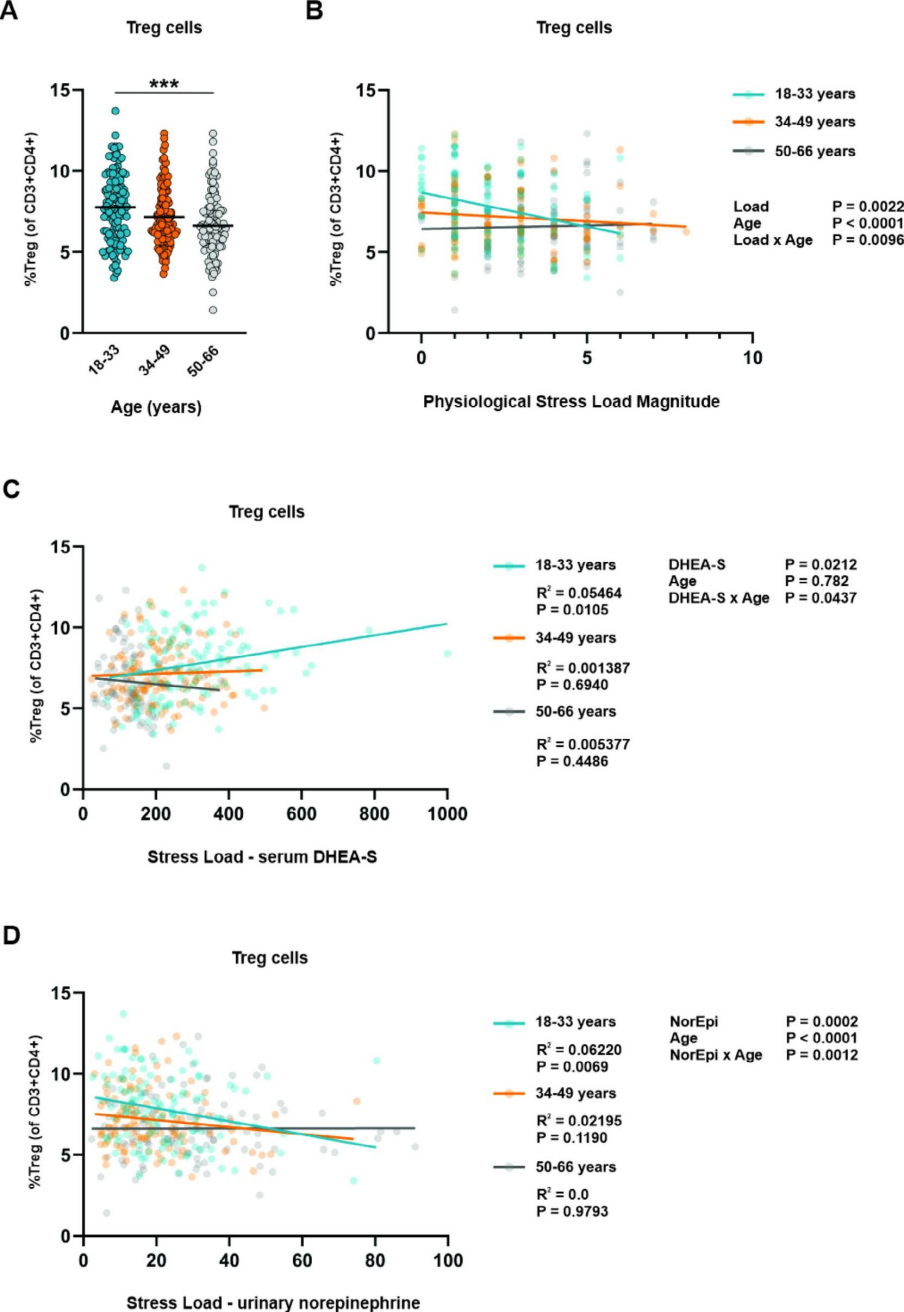
Association between physiological stress load and Treg frequencies. (**A**) Frequency of Tregs grouped by subject age. Statistical analysis was performed using Kruskal-Wallis non-parametric one-way ANOVA; ***P < 0.001. Linear regression of Treg frequencies and (**B**) physiological stress load, (**C**) serum DHEA-**S**, and (**D**) urinary norepinephrine for young (18–33 y), middle (34–49 y), and old (50–66 y) subjects. Correlation squared (R^2^) and *P* values for each association constant are shown for the linear regression

Next we examined the relation between physiological stress load and age-associated changes in circulating lymphocytes including naïve and central/effector memory CD4 and CD8 T cells, Th1, Th2, Th17 cells, NK cells and B cells, as well as total, naïve and memory Treg cells. For Tregs, defined as CD3 + CD4 + CD25 + CD127^low^, which comprised significantly smaller proportions of circulating lymphocytes in adults 50–66 y (Fig. 2A), we found a statistically significant (P = 0.0096) stress load by age interaction. This suggests an age-dependent association between physiological stress load and Treg frequencies. As shown in Fig. 2B, compared to participants 34–49 y or 50–66 y, Treg frequencies were higher in younger participants, but only in participants exhibiting low physiological stress load. As stress load increased, the frequency of Tregs dropped in young participants (β = -0.42 ± 0.12; r2 = 0.10; P = 0.0010) but were unchanged with increasing stress load in middle (P = 0.2711) and older (P = 0.6882) age individuals. Follow-up analysis of individual stress load components suggest circulating DHEA-S (Fig. 2C) and urinary norepinephrine (Fig. 2D) likely contribute to the age-dependent association between total stress load and Treg frequencies. Of all components tested, we only found a significant interaction between DHEA-S and Treg frequencies, and urinary norepinephrine and Treg frequencies. As with total stress load, only in young participants did we find significant associations between these physiological variables and frequencies of Tregs, with DHEA-S having a positive association with Treg populations (β = 0.004 ± 0.001; r2 = 0.05; P = 0.0105) and norepinephrine having a negative association with Treg populations (β= -0.04 ± 0.01; r2 = 0.06; P = 0.0069).

### Frequencies of naïve and memory regulatory T lymphocytes and CD4 T cells are associated with physiological stress load independent of subject age

**Fig. 3:**
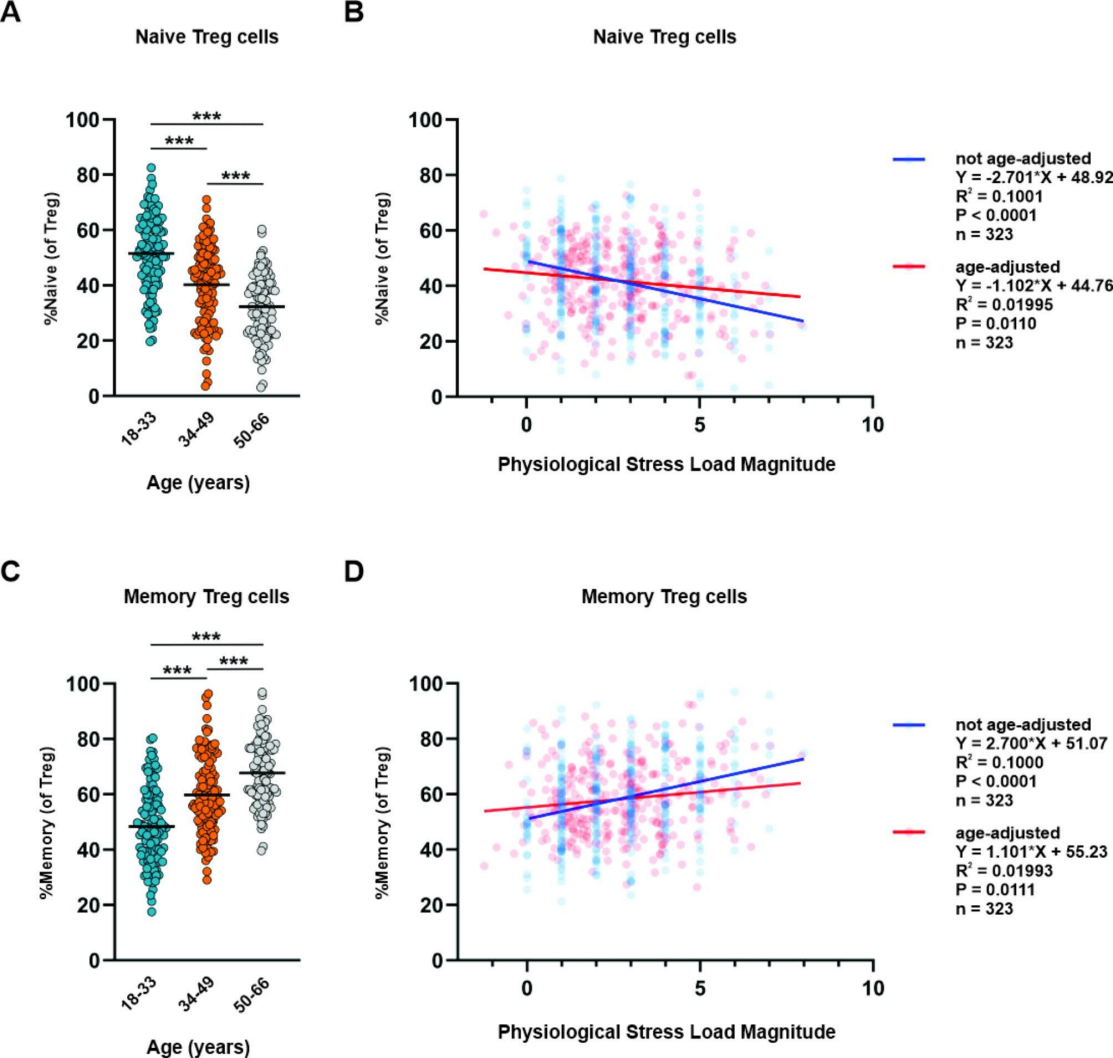
Association between physiological stress load and naïve and memory Tregs. Frequency of (**A**) naïve Tregs and (**C**) memory Tregs grouped by subject age. Statistical analysis was performed using Kruskal-Wallis non-parametric one-way ANOVA; ***P < 0.001. Not age-adjusted and age-adjusted linear regression of (**B**) naïve Treg frequencies and (**D**) memory Treg frequencies and physiological stress load. Equation, correlation squared (R^2^), *P* value for the association constant, and number of subjects are shown for each linear regression

Older participants (50–66 y) exhibited smaller frequencies of CD45RO^low^ naïve Tregs and naïve CD4 T cells but greater frequencies of CD45RO^hi^ memory Tregs and memory CD4 T cells compared to adults 18–33 y and 34–49 y (Fig. 3A, 3C and Supplementary Fig. 1A, 1C). After statistically controlling for age, we found that frequencies of these cell populations could partly be explained by variability in physiological stress load. We did not observe a stress load by age interaction, suggesting that these age-adjusted relationships between stress load and naïve and memory T cell frequencies did not depend on age in our study population. Increases in stress load associated with lower frequencies of naïve Tregs (Fig. 3B) and naïve CD4 T cells (Supplementary Fig. 1B) and higher frequencies of memory Tregs (Fig. 3D) and memory CD4 T cells (Supplementary Fig. 1D). The linear relationships between physiological stress load and frequencies of naïve and memory Tregs as well as naïve and memory CD4 T cells with and without age included in the statistical models are presented in Fig. 3 and Supplementary Fig. 1 respectively. Stress load components most likely contributing to linear relationships between physiological stress load and frequencies of naïve and memory Tregs include systolic and diastolic blood pressure and waist circumference while components most likely contributing to linear relationships between physiological stress load and frequencies of naïve and memory CD4 T cells include waist circumference, CRP, and HbA1c (Table 2). Together, these results indicate that stress load significantly associated with the frequencies of circulating naive and memory Tregs, even after statistically controlling for age effects. However, it is also clear that considering both stress load and age in the model explains more variability in the observed person-to-person differences in naïve and memory Treg lymphocytes.

**Table 2:**
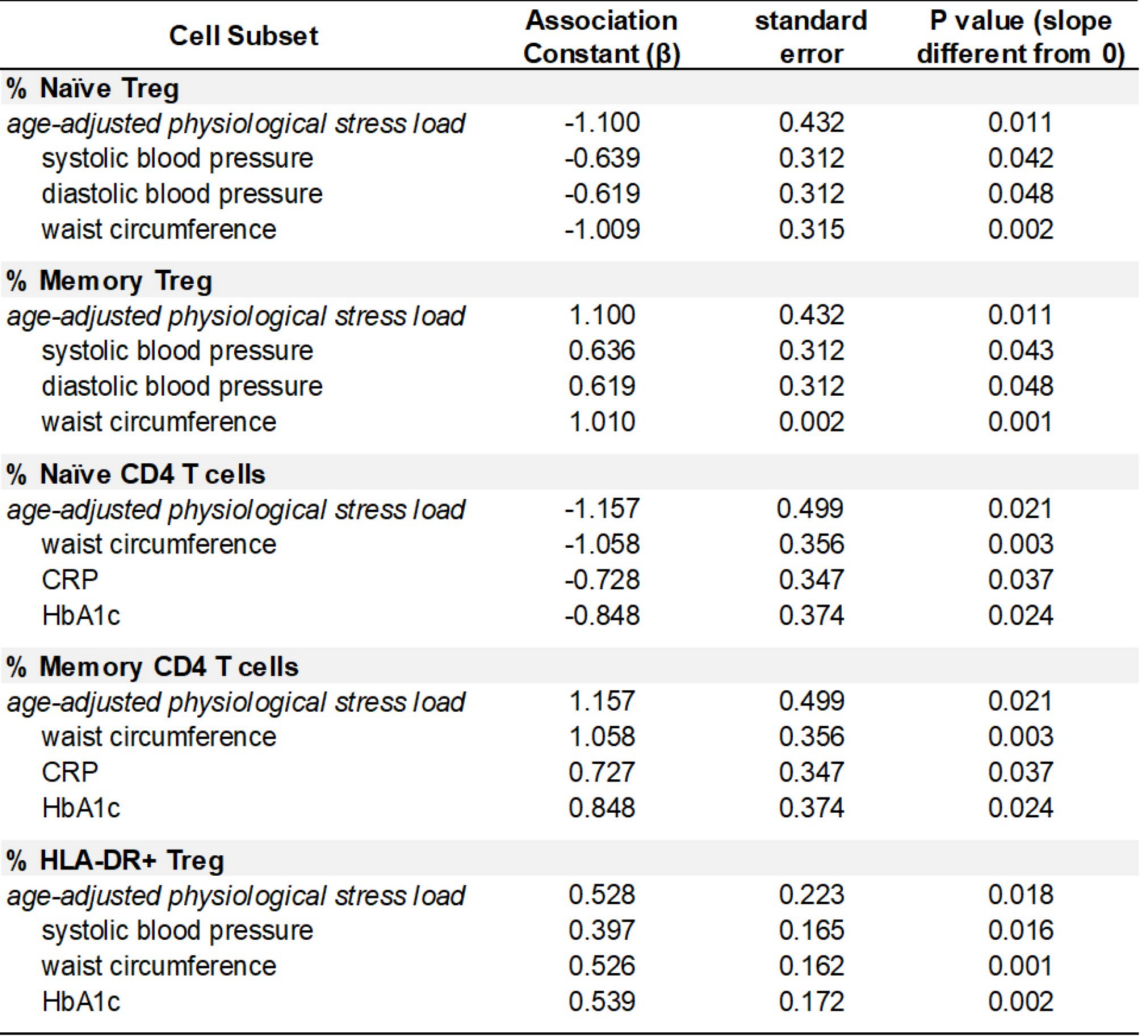
Association constants of physiological stress load parameters. Association constants, standard errors, and *P* values for relationships between cell frequencies and age-adjusted physiological stress load parameters

### Relation of physiological stress load with HLA-DR + regulatory T lymphocytes

**Fig. 4:**
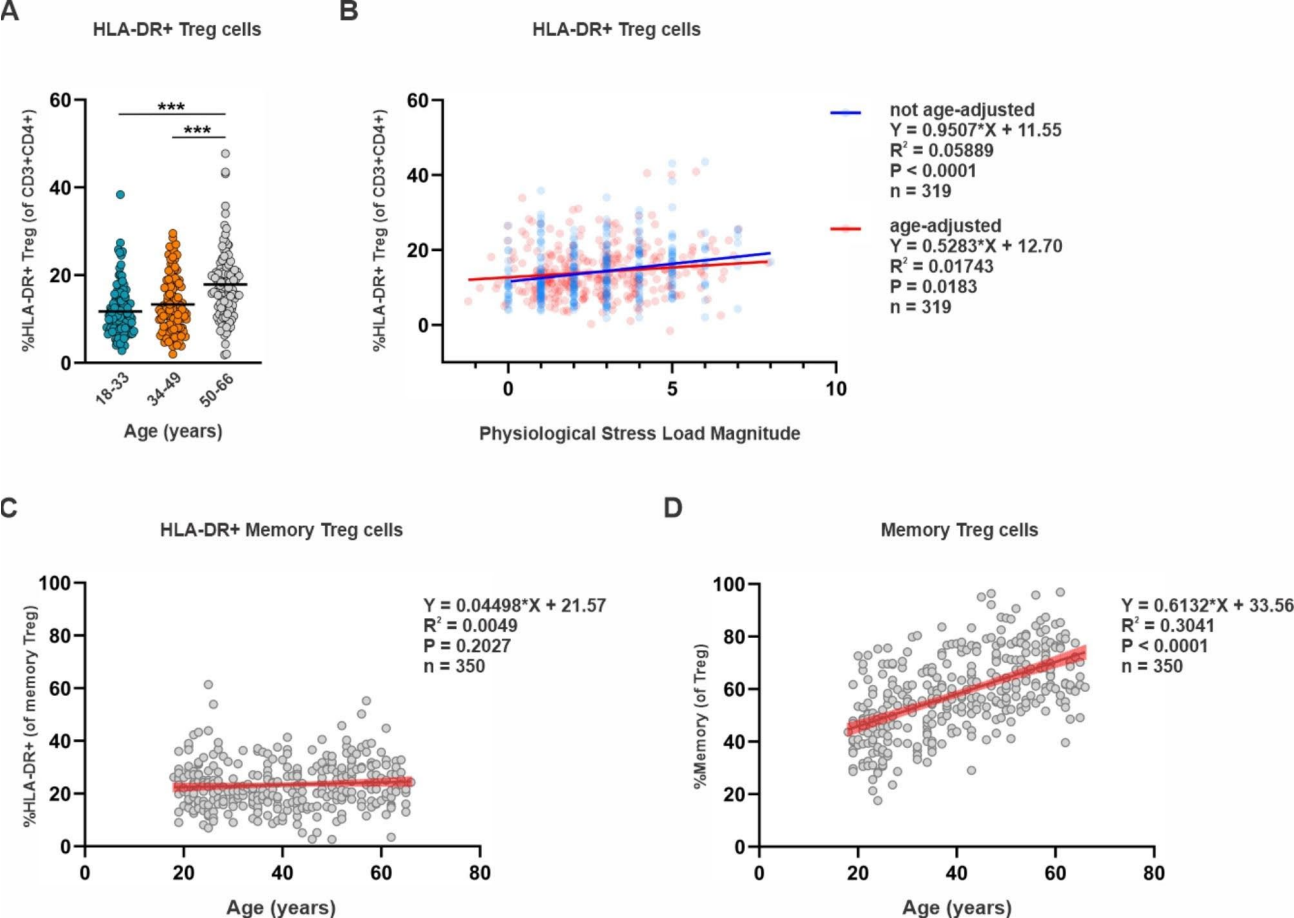
Association between physiological stress load and frequencies of HLA-DR + Tregs. (**A**) Frequency of HLA-DR + Tregs grouped by subject age. Statistical analysis was performed using Kruskal-Wallis non-parametric one-way ANOVA; ***P < 0.001. (**B**) Not age-adjusted and age-adjusted linear regression of HLA-DR + Treg frequencies and physiological stress load. Relationships between subject age and (**C**) HLA-DR + memory Tregs and (**D**) memory Tregs. Black lines indicate the linear line of best fit and red indicates the 95% confidence bands of the best-fit line. Equation, correlation squared (R^2^), *P* value for the association constant, and number of subjects are shown for each linear regression

HLA-DR expression on regulatory T cells identifies a functionally distinct and highly immunosuppressive subset of Tregs [21, 22]. As shown in Fig. 4A, older individuals exhibited significantly higher frequencies of HLA-DR + Tregs. Results also demonstrate the relationship between stress load and frequency of HLA-DR + Tregs with and without statistically controlling for age. Even after controlling for effects of age, stress load independently associated (P = 0.0174; r2 = 0.02) with HLA-DR + Treg populations (Fig. 4B). This observation suggests that similar to increasing age, greater levels of stress load independently associate with a higher proportion of HLA-DR + Tregs. With respect to frequencies of HLA-DR + Tregs, we did not find a stress load by age interaction. As shown in Table 2, the stress load components most likely contributing to the physiological stress load association with HLA-DR + Treg frequencies include systolic blood pressure, waist circumference and HbA1C. Our results also show that while HLA-DR + memory Treg frequencies remain stable with age (Fig. 4C), frequencies of memory Tregs significantly increase with age (Fig. 4D). Together this implies that increased frequencies of HLA-DR + Tregs observed in older subjects is due in part to the expansion of the memory Treg pool comprised of HLA-DR + Treg cells. This finding is also supported by our physiological stress load association data showing frequencies of memory Tregs and HLA-DR + Tregs both positively associate with systolic blood pressure and waist circumference (Table 2).

## Discussion

To investigate relationships between cumulative physiological stress and changes in adaptive immune cell populations in a cohort of healthy adult volunteers ranging in age and recruited to a cross-sectional Nutritional Phenotyping Study [14, 25], we calculated a composite measure of each subject’s cumulative physiological stress load using a battery of biomarkers devised by Seeman and McEwen [8–11]. In alignment with previous reports [23, 24], our data showed physiological stress load positively correlated with subject age and was significantly higher in adults 50–66 y compared to adults 18–33 y and 34–49 y. Using covariate analysis we identified a significant age-dependent association between physiological stress load and frequencies of circulating Tregs. Results showed frequencies were higher in younger participants, but only in participants exhibiting low physiological stress load. As stress load increased, the frequency of Tregs decreased in young participants but were unchanged with increasing stress load in middle and older age individuals. Increasing stress load in young participants reduced Treg frequency to levels seen in middle and older aged participants. Analysis indicated circulating DHEA-S and urinary norepinephrine contributed to the age-dependent association between total stress load and Treg cells, suggesting frequencies of Tregs are sensitive to stress (norepinephrine) and anti-stress (DHEA-S) physiological mediators in younger people, but less so in older individuals. In older individuals, other age-associated factors may better explain the age-related changes in circulating frequencies of Tregs.

Steroid hormone DHEA, and its sulfated form DHEA-S, are abundantly secreted from the adrenal gland [25]. In contrast to glucocorticoids, DHEA and DHEA-S exert immunostimulatory properties [26, 27]. DHEA-S is converted to biologically active DHEA which epigenetically regulates Tregs to increase Foxp3 expression through androgen receptor (AR) activation [28]. In addition, DHEA-mediated AR activation was shown to stabilize Treg suppressive function in a murine model of allergic airway inflammation [29]. Decreased DHEA-S serum concentrations in humans are also associated with autoimmune pathologies involving Tregs including rheumatoid arthritis [30] and systemic lupus erythematosus [31]. The association of Treg frequencies with levels of DHEA-S in young subjects but less so in older individuals is also intriguing considering circulating levels of DHEA-S peak around ages 25–30 then decline sharply in an age-dependent manner [26]. While the secretion of DHEA has been shown to have several effects on the human body including reducing inflammation and improving sexual and cognitive function [26], its immune-modulating properties remain poorly understood. In contrast to DHEA-S, levels of norepinephrine inversely associated with Treg frequencies in young subjects. Norepinephrine, a catecholamine neurotransmitter secreted by local sympathetic neurons and adrenal medulla, modulates a variety of immune cell functions through activation of adrenergic receptors [32]. In mice, norepinephrine reduced frequencies of Foxp3 positive cells and expression of Foxp3 mRNA via β2-adrenoceptor-mediated mechanisms [32] while in humans, catecholamines including norepinephrine have been shown to suppress Treg functions [33].

How aging impacts Treg frequencies in peripheral blood remains unclear. Although we observed reduced frequencies of Tregs with age in our cohort of more the 300 subjects, not all studies have reported similar findings. In animal studies, Treg production was shown to decline more and faster than conventional T cells while differentiation of naïve conventional T cells into peripheral Tregs was lower in aged mice compared to their young counterparts [34, 35]. While Miyara et al. [36] reported reduced frequencies of total Tregs in aged human donors, other studies have either failed to detect changes between young and old subjects [37, 38] or reported increased frequencies in older subjects [39, 40]. Such discordances between studies may stem from different phenotypic markers used to define Treg populations such as CD3 + CD4 + CD25 + CD127low [41–44], CD4 + FoxP3+ [38, 40], and CD4 + CD25 + FoxP3+ [45]. While in mice FoxP3 is an almost exclusive marker of Tregs [46], human FoxP3 + T cells are more heterogenous and include naïve and effector Tregs as well as non-Tregs [21, 47, 48]. For this reason, we defined Tregs as CD3 + CD4 + CD25 + CD127low because these cells have been shown to be highly suppressive [43, 44], express high levels of FoxP3 [43, 44], correlate strongly with frequencies of CD4 + CD25 + FoxP3 + T cells in human blood [38, 44] while also excluding FoxP3 + non-Tregs [43, 44]. An additional classification of human Tregs based on expression levels of CD45RA and FoxP3 has been proposed in which FoxP3 + CD4 + T cells are divided into three fractions; naïve Tregs (CD45RA + FoxP3lowCD4+), effector Tregs (CD45RA–FoxP3highCD4+), and non-Tregs (CD45RA–FoxP3lowCD4+) [49]. Using this strategy, it was found that the proportion of naïve Tregs was decreased in aged donors versus younger donors while that of effector Tregs and non-Tegs were increased in aged donors versus younger donors [36]. Additional studies incorporating various Treg phenotypic markers will help clarify the impact of physiological stress on populations of circulating Treg.

With advanced age and reduced thymic output, maintenance of the naïve T cell pool relies more heavily on peripheral division of existing clones [2]. With time this results in a smaller pool of naïve and a larger pool of memory cells. Consistent with this paradigm, older participants (50–66 y) in our cohort exhibited smaller frequencies of naïve Tregs and naïve CD4 T cells but greater frequencies of memory Tregs and memory CD4 T cells compared to younger adults. After statistically controlling for age, we found increased physiological stress load associated with lower frequencies of naïve Tregs and naïve CD4 T cells and higher frequencies of memory Tregs and memory CD4 T cells. Waist circumference and systolic and diastolic blood pressure were **s**tress load components contributing most to the age-associated shift from naïve to memory Tregs while waist circumference, CRP, and HbA1c contributed most to the age-related shift from naïve to memory CD4 T cells in our study cohort.

In summary, our results show that physiological stress load is associated with age-related changes in circulating Treg populations in healthy adult volunteers. While follow-up studies are clearly needed to determine causative effects of physiological stress load factors such as DHEA-S, norepinephrine, and blood pressure on Treg cells, our findings suggest that biological responses to chronic environmental, physiological, and psychosocial stressors may accentuate the age-related reduction of Treg frequencies in younger people and also contribute to contraction of the naïve Treg pool and accumulation of memory Treg cells.

## Electronic supplementary material

Below is the link to the electronic supplementary material.

Supplementary Material 1: **Fig. 1**. Association between physiological stress load and naïve and memory CD4 T cells. Frequency of (A) naïve CD4 T cells and (C) memory CD4 T cells grouped by subject age. Statistical analysis was performed using Kruskal-Wallis non-parametric one-way ANOVA; ***P < 0.001. Not age-adjusted and age-adjusted linear regression of (B) naïve CD4 T cell and (D) memory CD4 T cell frequencies and physiological stress load. Equation, correlation squared (R^2^), *P* value for the association constant, and number of subjects are shown for each linear regression.

Supplementary Material 2: **Table 1**. Antibodies used in flow cytometry panels.

## Data Availability

Requests for data from the USDA ARS WHNRC Nutritional Phenotyping Study used in this analysis should be made via an email to the senior WHNRC author on this publication. Requests will be reviewed quarterly by a committee consisting of the study investigators.

## Abbreviations

AL: Allostatic load
CRP: C-Reactive Protein
DHEA: Dehydroepiandrosterone
HPA: Hypothalamic-pituitary-adrenal
SNS: Sympathetic nervous system
Treg: Regulatory T lymphocyte
