# Supplementary figures and images for: Cumulative physiological stress is associated with age-related changes to peripheral T lymphocyte subsets in healthy humans
Source: Immun Ageing. 2023 Jun 23;20:29. doi: 10.1186/s12979-023-00357-5 (PMC10288764; doi:10.1186/s12979-023-00357-5)

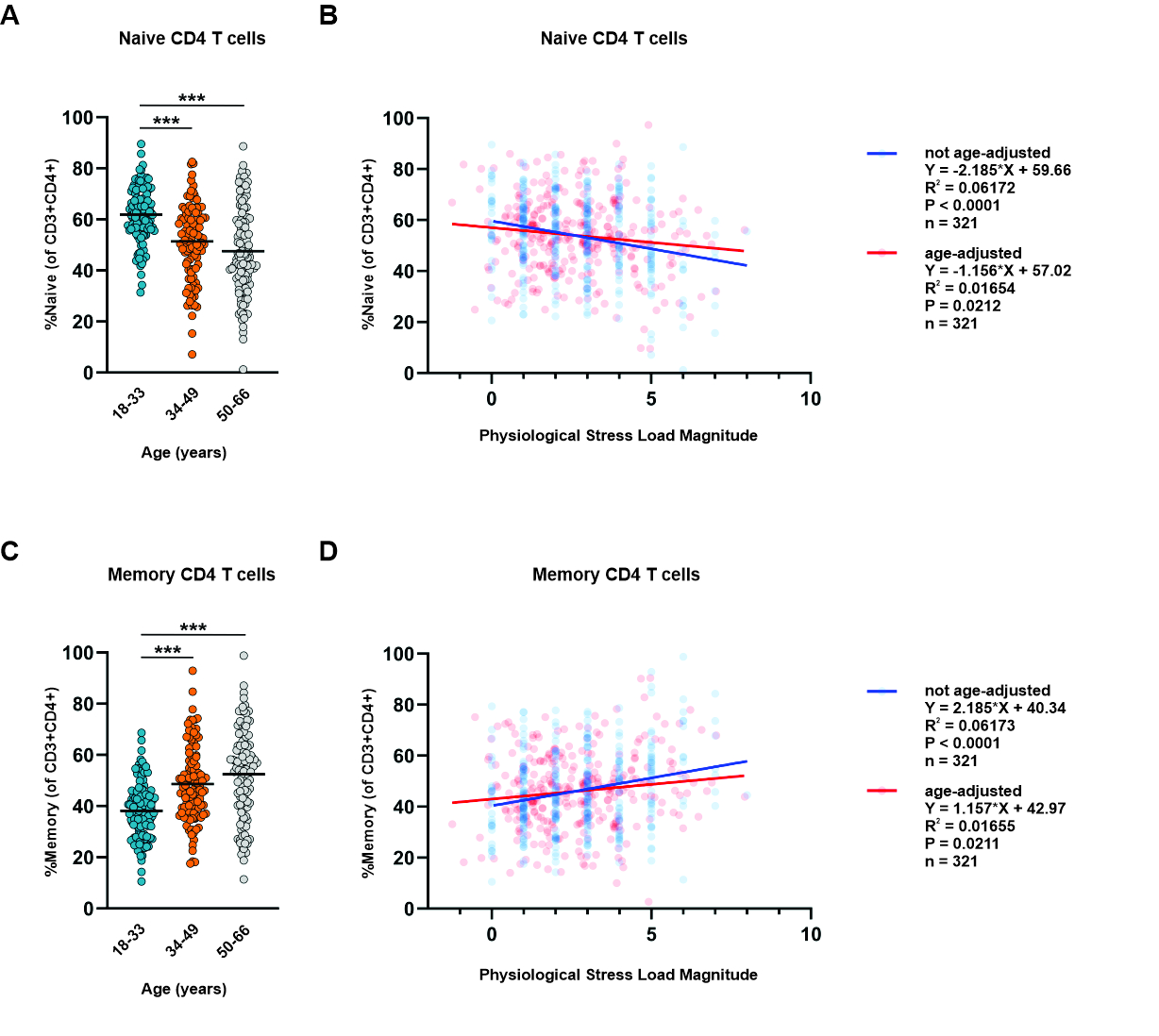

Supplement: Supplementary file 1 — Supplementary Material 1: Fig. 1. Association between physiological stress load and naïve and memory CD4 T cells. Frequency of (A) naïve CD4 T cells and (C) memory CD4 T cells grouped by subject age. Statistical analysis was performed using Kruskal-Wallis non-parametric one-way ANOVA; ***P < 0.001. Not age-adjusted and age-adjusted linear regression of (B) naïve CD4 T cell and (D) memory CD4 T cell frequencies and physiological stress load. Equation, correlation squared (R2), P value for the association constant, and number of subjects are shown for each linear regression. [file 12979_2023_357_MOESM1_ESM.jpg]
