# Supplementary material for: Cumulative physiological stress is associated with age-related changes to peripheral T lymphocyte subsets in healthy humans
Source: Immun Ageing. 2023 Jun 23;20:29. doi: 10.1186/s12979-023-00357-5 (PMC10288764; doi:10.1186/s12979-023-00357-5)
Supplement: Supplementary file 2 — Supplementary Material 2: Table 1. Antibodies used in flow cytometry panels. [file 12979_2023_357_MOESM2_ESM.docx]

**Supplementary Table 1. Antibodies used in flow cytometry panels.**
